# Ultra structural changes occurring in duct ectasia and periductal mastitis and their significance in etiopathogenesis

**DOI:** 10.1371/journal.pone.0173216

**Published:** 2017-03-08

**Authors:** Kirithiga Ramalingam, Seenu Vuthaluru, Anurag Srivastava, Amit Kumar Dinda, Anita Dhar

**Affiliations:** 1 Department of Surgical Disciplines, All India Institute of Medical Sciences, New Delhi, India; 2 Department of Pathology, All India Institute of Medical Sciences, New Delhi, India; Fu Jen Catholic University, TAIWAN

## Abstract

**Introduction:**

Duct ectasia (DE) and periductal mastitis (PDM) are the most common benign breast conditions seen in women. The etiopathogenesis of these entities is still not clear and most of the theories regarding the causation are based on the histological features as seen on light microscopy. The ultramicroscopic features associated with these conditions that may give more insight to the etiopathogenesis are unknown.

**Aim:**

To study the ultrastructural changes occurring in mammary duct cones in patients with DE and PDM using Transmission Electron Microscopic (TEM).

**Method:**

Major ducts removed by radical duct excision from 21 patients with final histopathological diagnosis of DE and PDM were subjected to TEM study with 2 normal duct samples as controls.

**Results:**

The TEM features of DE were denudation of the epithelial cells with focal loss of microvilli, widening of the inter-epithelial junctions with focal disruption of the T bars, periductal collagenisation without inflammation, and features suggestive of Epithelial Mesenchymal Transition (EMT). PDM features are intact epithelial lining with proliferative epithelium and periductal collagenisation with inflammation.

**Conclusions:**

Based on the TEM findings, we suggest that DE and PDM are two different entities. EMT a novel finding observed in DE in this study.

## Introduction

Nipple discharge among women is a common clinical entity. Duct ectasia (DE) / periductal masititis (PDM) is an important cause of this clinical condition. In spite of the enormous research, the etiopathogenesis and the structural pathological changes that take place in this condition are largely unknown. Literature is not clear as to whether DE and PDM are two different entities or different manifestations of a common clinical condition. A study of the sub cellular changes of DE and PDM may throw more light on these issues and may also provide insight into a specific target for local therapy if any, which is lacking so far.

## Objective

To evaluate the ultra structural changes associated with DE and PDM by Transmission electron microscopic (TEM) study of mammary duct cones of patients with DE/PDM.

## Materials and methods

Forty one consecutive patients with non bloody nipple discharge with clinical and radiological features suggestive of DE/PDM presenting to the surgical outpatient department of All India Institute of Medical Sciences, New Delhi were included in the study after obtaining Institute ethics committee’s approval and informed, written consent. Patients who had the following presentations were diagnosed to have duct ectasia- Presence of thick, colored, or creamy nipple discharge, thickened subareolar ducts, nipple retraction, and absence of signs of inflammation with sonographic features of dilated subareolar ducts and mammographic features of prominent ducts appearing as conical opacities with apex of the cone towards the nipple with or without characteristic coarse calcifications (ring like, lying on the duct wall or circular or needle shaped when the duct contents are calcified). Those who had history suggestive of previous episodes of subareolar sepsis, presence of pus/serous nipple discharge, and signs of periductal inflammation, tender palpable subareolar ducts, nipple retraction, and mammary fistula with radiological features of dilated ducts and subareolar abscess were considered to have periductal mastitis. Excluded from the study were those patients who were unwilling to give consent, women with bloody nipple discharge and cytopathology of nipple discharge smear demonstrating atypical or malignant cells. A detailed history and thorough physical examination were done for all patients along with ultrasound of both the breasts and axillae. For women who were above 35 years digital ammography was done. Cytopathological examination of the nipple discharge smear was carried out in patients with active nipple discharge. Patients who had active subareolar sepsis were treated with antibiotics prior to the surgery. Major mammary duct excision, the surgical procedure of choice was performed in all [S1 File]. The excised ductal tissues were processed for both light and electron microscopy. The histopathological features for making the diagnosis of duct ectasia were 3 or more dilated ducts with thickened or sclerosed duct walls, periductal sclerosis, hyperelastosis with or withoutepithelial denudationand no or minimal inflammation. The histopathological features for reaching the diagnosis of periductal mastitis were dense periductal inflammation and fibrosis with or without focal dilatation of ducts. Twenty patients who had pathological diagnoses of mixed duct ectasia/periductal mastitis with fibrocystic disease (n = 12), papilloma (n = 3), papillomatosis (n = 3) and tuberculous mastitis (n = 2) were excluded from the electron microscopic study. Based on the final histopathological diagnoses, 21 patients with definitive diagnoses of pure DE (n = 9), PDM (n = 7) or mixed features of DE and PDM i.e. duct ectasia with features of peridcutal inflammation (n = 5) were included in the TEM study [S2 File]. The tissues were fixed in 2.5% glutaraldehyde and processed by automatic tissue processor. Several resin sections were cut at approximately 1 micron using glass knives made by the knife maker and an ultra microtome. The ultrastructural examination was performed on Philips Morgagni 268 transmission electron microscope and Technai G20 transmission electron microscope. The normal mammary ducts sampled from 2 patients who underwent reduction mammoplasty were taken as control. These two ladies did not suffer from any nipple discharge and were premenopausal with regular menstrual cycles.

## Results

**Fig 1:**
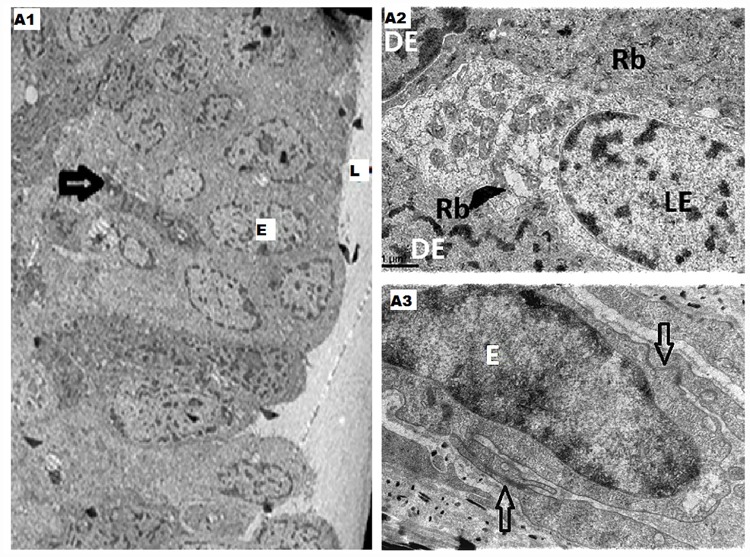
A1- This electro micrograph shows transverse section of the major duct with continuous epithelial layer [E] with underlying myoepithelial cells [denoted by arrow] which are discontinuous. L—Lumen of the duct. [x570]; A2- The above electro micrograph is a higher magnification of dark [DE] and light epithelial cells [LE], with dark epithelial cells showing dense distribution of ribosomes [Rb]. A3- This electro micrograph of a duct in the patient shows the presence of T bars in the inter-epithelial junctions [as depicted by the two arrows]. E- epithelial cell, L- lumen of the duct. [x5000].

TEM study revealed the following features. The normal major mammary ducts showed a continuous epithelial layer (single or double layered) and myoepithelial cells interspersed in a discontinuous manner [Fig 1-A1]. Two types of epithelial cells lining the major mammary duct could be appreciated namely, the dark epithelial cells, being rich in electron dense ribosomes and the light epithelial cells [Fig 1-A2]. In the interepithelial junctions there were terminal bars [T bars] which were electron dense [Fig 1-A3]. The above mentioned findings correlated with the observations and the images documented in the TEM study of normal mammary ducts done by the various authors in the literature[1–4].

**Fig 2:**
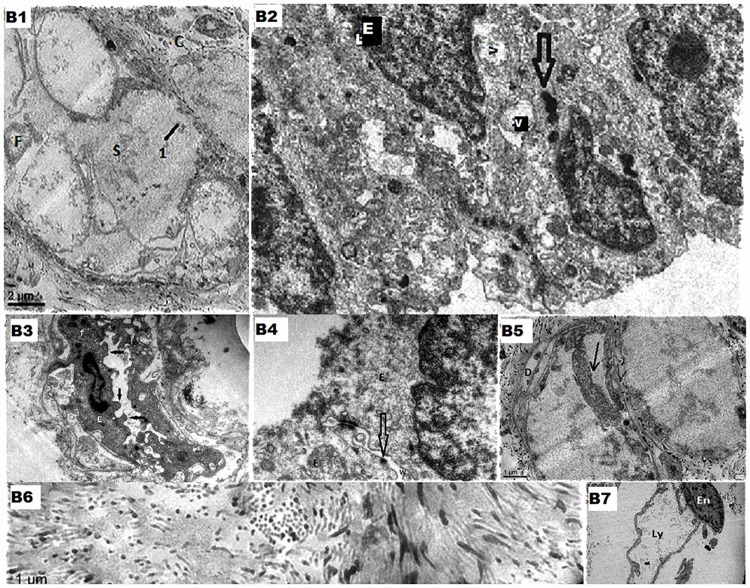
B1- Low power electro micrographic view of a dilated duct with periductal tissue showing focal denudation [1] of lining epithelium with intraluminal secretions[S] containing epithelial fragments [F]. Around the duct there is collagen deposition. [x1100]; B2- The epithelial cells in this show large number of vacuoles [V] and dense bodies [indicated by the arrows]. E- epithelial cell. [x5000]; B3- The duct epithelial cell [E] shown in the electro micrograph has elongated nucleus, intracytoplasmic fibrils [f] and intraluminal projections [indicated by the arrows] of the cytoplasm suggestive of mesenchymal transformation of the epithelial cell. [x3200]; B4- This electro micrograph depicts focal distortion of terminal bar [indicated by the arrow] and widening of the interepithelial junction [W]. E –epithelial cells. [x8000]; B5- This electro micrograph shows lifting of the epithelium from the basal lamina [arrow] and duplication of the basal lamina[D]. [x2550]; B6- The above electro micrograph shows periductal tissue filled with thick and thin collagen fibres indicative of active collagenisation. [x2550]; B7- Dilated lymphatic vessel [Ly] with the endothelial cell [En] in the periductal tissue seen in this electro Micrograph. [x1100].

In DE, there is flattening of the epithelium in the dilated duct in some areas with focal loss of microvilli of the duct epithelium & accumulation of intraluminal epithelial debris (seen in 9/14 (64.3%)). [Fig 2-B1]. While the cytoplasm revealed multiple membrane bound dark and light vesicles, [Fig 2-B2], and plenty of intracytoplasmic fibrillary structures, the nuclei appeared elongated—features suggestive of epithelial mesenchymal transition [EMT] were observed in 8/14 patients (57.1%) [Fig 2-B3]. T bars showed focal distortion [Fig 2-B4] with widening of the inter-epithelial junction were seen in 7/14 cases (50%) [Fig 2-B5]. Small ducts (ductules) [Fig 2-B6] showed similar epithelial changes as that of the major ducts with duplication of the basal laminae being observed in 7/14 patients (50%). Surrounding connective tissues showed marked periductal collagenisation (11/14 cases i.e. in 78.6%) with dilated lymphatic vessels [Fig 2-B7].

**Fig 3:**
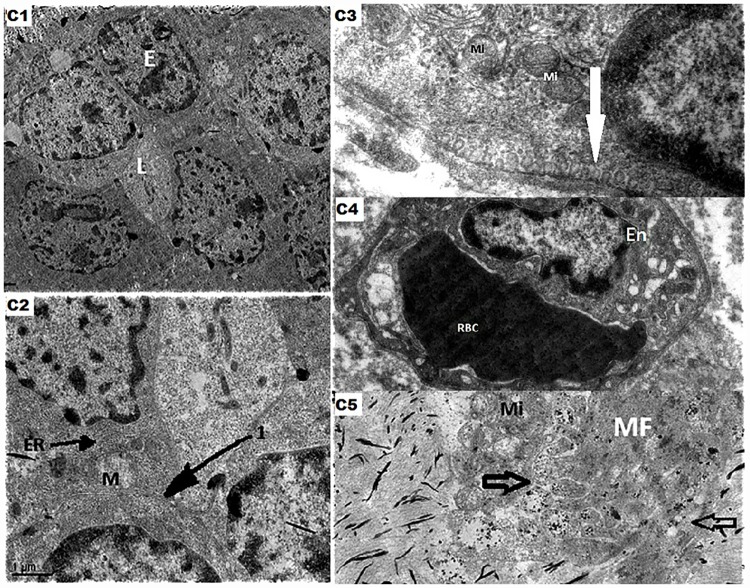
C1- The above mentioned electro micrograph shows transverse section of the duct lined by hypertrophic epithelial cells [E] almost filling the entire lumen [L] indicative of epithelial proliferation. BL—Basal Lamina. [x1100]; C2- The arrow in the above mentioned electro micrograph represents the inter epithelial junction. The epithelial cell shows dilated mitochondria with loss of cristae [M] and prominent endoplasmic reticulum [ER]. [x2550]; C3- The ultrastructure of the portion of the duct epithelial cell depicted in this electro micrograph shows numerous pinocytic vesicles [some fusing with the membrane—depicted by the arrow] suggestive of active secretion by the epithelial cells. The cytoplasm also shows large mitochondria with dilated cristae. [x13000]; C4- This electro micrograph shows capillary in the periductal tissue with plump endothelial cell and an inspissated red blood cell. This is suggestive of neovascularisation secondary to inflammation. [x3200]; C5- This electro micrograph shows a portion of a myofibroblast cell[MF] with lot vesicles [indicated by the arrows] containing collagen granules suggestive of active collagenisation. The cell also shows multiple dilated mitochondriae [Mi]. All these changes are indicative of exaggerated cell function.C—Collagen fibres. [x1550].

In PDM, the epithelial cells were hypertrophic indicating active proliferation in 3 out 7 cases (42.9%) [Fig 3-C1]. The epithelial cell cytoplasm showed dilated mitochondriae with loss of cristae and prominent endoplasmic reticuluae suggestive of injury in 5 patients (71.4%) [Fig 3-C2]. There were numerous pinocytic vesicles in the cytoplasm of the epithelial cells suggestive of active secretion by the duct epithelial cells [Fig 3-C3]. However, unlike in duct ectasia, the inter-epithelial junctions were intact with preserved T bars in all the cases of PDM (100%). Periductal tissue showed neo-vascularisation secondary to inflammation in the periductal tissue in 3 patients (42.9%) [Fig 3-C4] & the fibroblast cells showed plenty of vesicles with abundant collagen granules suggestive of active collagenisation in all the cases (100%) [Fig 3-C5].

**Fig 4:**
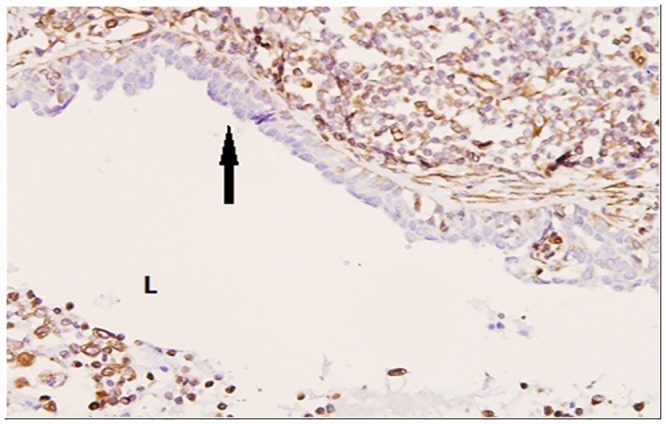
The arrow in the photomicrograph indicates the focal uptake of vimentin by the epithelial cells. L- Lumen of the duct.

Immunohistochemistry [IHC] for Vimentin [A mesenchymal marker]: IHC for vimentin was done in 4 cases of PDM and 5 cases of DE. The epithelial cells in all the 5 cases of DE have showed focal areas of uptake of vimentin, which was not seen in cases of PDM [Fig 4]. This finding again supported the EMT which was observed on the TEM study of DE specimens.

## Discussion

There is no data available on the electron microscopic study of DE/PDM in literature. Hughes et al [5] have commented that there is no loss of microvilli in scanning electron microscope in cases of DE. However, no further details have been mentioned. Scanning electron microscopy as used by Hughes et al [5]describes only the surface details while TEM describes in detail about the intracellular changes which helps us to understand the pathogenetic mechanisms better. In DE, the changes observed suggested EMT. EMT is an interesting biological phenomenon, by which epithelial cells get transformed into mesenchymal cells and acquire new characteristics such as motility and collagen synthesis. The EMT in duct ectasia has been substantiated by demonstration of vimentin positive cells among the normal epithelial cells in patients with DE and presence of epithelial cells with elongated nuclei and cytoplasmic processeses containing fibril like material. Thus EMT seems to may play a key role in the pathogenesis of DE. Though periductal collagenisation is seen both in DE and PDM, in PDM, the collagenisation is associated with inflammation unlike in DE. Thus, it is evident that DE and PDM may be considered as two different entities with two different pathogenetic mechanisms.

The etiopathogenesis of DE and PDM are poorly understood. The classical concept as enunciated by Haagensen and Ewing [6] considers duct dilatation under hormonal influence as a primary event. This leads to epithelium ulceration, followed by leakage of secretion into the periductal tissue leading to inflammation, secondary bacterial infection and periductal fibrosis. An alternative explanation by Dixon et al considers DE and PDM as two different entities & PDM does not precede DE.

These controversies can be addressed by the ultramicroscopic features of DE and PDM observed in the present study. Distinct changes in the epithelial lining and in periductal tissue in DE and PDM were observed on electron microscopy and immuno-histochemistry. In this study, pure DE was seen in nine patients, pure PDM in six patients and a combination of both DE and PDM i.e. DE with periductal inflammation in six patients. The combination of both being present together could be explained by the fact that there could be secondary duct dilatation following periductal fibrosis.

Identification of EMT changes in patients of DE in our study adds mammary ducts to the list of organs where in EMT seems to play a role in pathologically induced fibrosis [7–11]. However, the signaling pathway involved in EMT of DE is yet to be studied. Success of BMP-6 & 7 (inhibitors of EMT induced fibrotic process) in reversal of renal fibrosis in animal models [12, 13] is encouraging to conduct further research to identify the common switch of EMT in DE. Once the switch if any is identified, it may be turned off by molecular targeting and thuscure the disease.

Limited number of controls due to ethical reasons is a limitation of this study. However, the normal human major mammary duct ultrastructure that has been documented in the literature along with the present study controls aids us to compare the changes that have been observed in DE and PDM. Another limitation of the study which is common in any of the TEM study is that the small tissue dimension used may not be representative of the entire disease process.

## Conclusions

As the electron microscopic findings of DE and PDM are distinct from one another, these can be considered as two different entities. The association of EMT in DE is a novel finding of the present study.

## Supporting information

S1 File Surgical procedure. (DOCX)

S2 File Electron microscopy procedure. (DOCX)

S3 File Histopathology, electron microscopy and immunohistochemistry images. (DOCX)
