# Supplementary material for: Ultra structural changes occurring in duct ectasia and periductal mastitis and their significance in etiopathogenesis
Source: PLoS One. 2017 Mar 8;12(3):e0173216. doi: 10.1371/journal.pone.0173216 (PMC5342207; doi:10.1371/journal.pone.0173216)
Supplement: S1 File — (DOCX) [file pone.0173216.s001.docx]

**MAJOR MAMMARY DUCT EXCISION**

The procedure was done using the modified technique as described by **Srivastava et al**. The steps of the procedure were standardized before the study and each procedure was performed in similar fashion by a consultant surgeon well versed in the breast surgery.

All the fourty one operations were performed under local anaesthesia on day care basis in main operation theatres of AIIMS. Eutectic mixture of Lignocaine and Prilocaine (Prilox 5% cream) was applied on the nipple-areola complex (Figure 4.3), 45 minutes prior to the procedure to anaesthetise the intact areolar skin in order to alleviate the pain and anxiety caused by initial needle pricks while introducing local anaesthetic injections. Cefazolin (an injectable first generation cephalosporin) 2 gram was administered, intravenously 30 minutes prior to the procedure to prevent surgical site infection after intradermal sensitivity testing. 2% Chlorhexidine solution in alcohol was used for preoperative skin cleansing. 1% solution of Lignocaine without adrenaline and 0. 25% solution of Bupivacaine were used as local anaesthetic agents (figure 4.4).

Incision was placed exactly at the areolo-cutaneous junction to obtain the maximum cosmetic benefit (figure 4.5). Care was taken not to include more than one-third of the areolar circumference and incision preferably was placed in the lower one the NAC (Nipple Areola Complex) to give a better cosmetic result. The incision was deepened and dissection was stopped once the tough, white ductal tissue passing to the nipple was identified (figure 4.6). Subareolar tunnel was made around the ductal cone by blunt dissection using a curved haemostat working from each side (figure 4.7). The ductal tissue was grasped with a cross action towel clip and by moving the towel clip vertically the ductal cone was separated from the fatty breast parenchyma (figure 4.8). The sharp edge of the cross action towel clip and its ability to encircle the ductal cone without crushing it, allowed easy stripping of the ductal cone from the surrounding fibro fatty tissue. Care was taken to include all the ductal tissue within the grasp of the towel clip. The ductal cone was transected from the undersurface of the nipple using using surgical knife (figure 4.9). The deep end of the ductal cone was divided using bipolar electro cautery to minimize bleeding. The Under surface of the nipple was examined carefully by everting it on the tip of the index finger to ensure the division of all the ducts. The terminal portions of the ducts and lactiferous sinuses attached to the under surface of the nipple were pared using scissors (figure 4.10. The inverted position of the nipple after division of the ductal cone indicated incompleteness of the procedure and the undivided peripherally situated ducts were looked for and divided. After complete division of all the ductal tissue, the nipple assumed an everted position. After ensuring haemostasis, the cavity was washed thoroughly with normal saline. Closed suction drainage (8 Fr,MiniRomoVac,) was used in all the cases. Wound was closed with a subcuticular suture using 4-0 Poliglecaprone 25 (Monocryl, Ethicon ) (Figure:4.12). Occlusive dressing or a paraffin gauze and cotton pad dressing was applied. Patient was discharged after few hours of observation in the ward with prescription of 5 days of tablet erythromycin 500mg 6^th^ hourly and tablet tinidazole 400 mg 8^th^ hourly. The suction drain and the occlusive dressing were removed on the second post operative day following the surgery.

**SPECIMEN HANDLING AND MARKING**

The excised ductal cone was marked by a silk suture at the nipple end of the specimen (Figure: 4.10). This was helpful in orienting the specimen in pathology laboratory. After marking, the specimen was immediately put in 10% formaline solution for detailed pathological evaluation. After 24 hours of formalin fixation, 4 to 5 transverse sections of the ductal cone wre cut for paraffin block preparation.


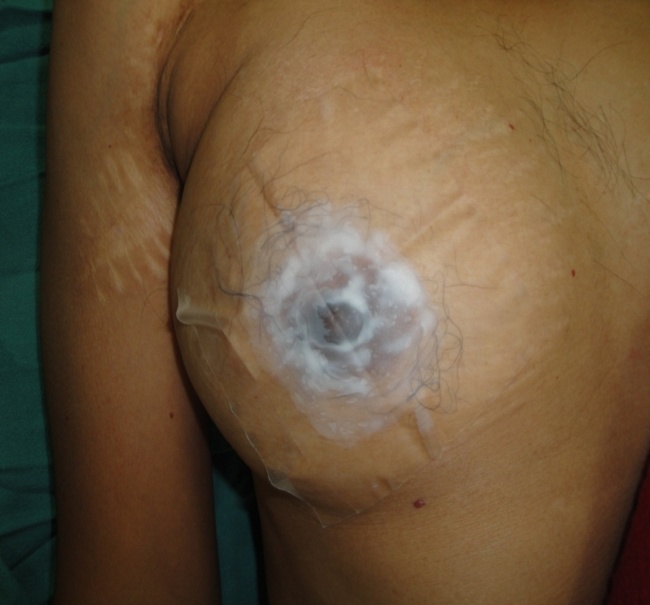

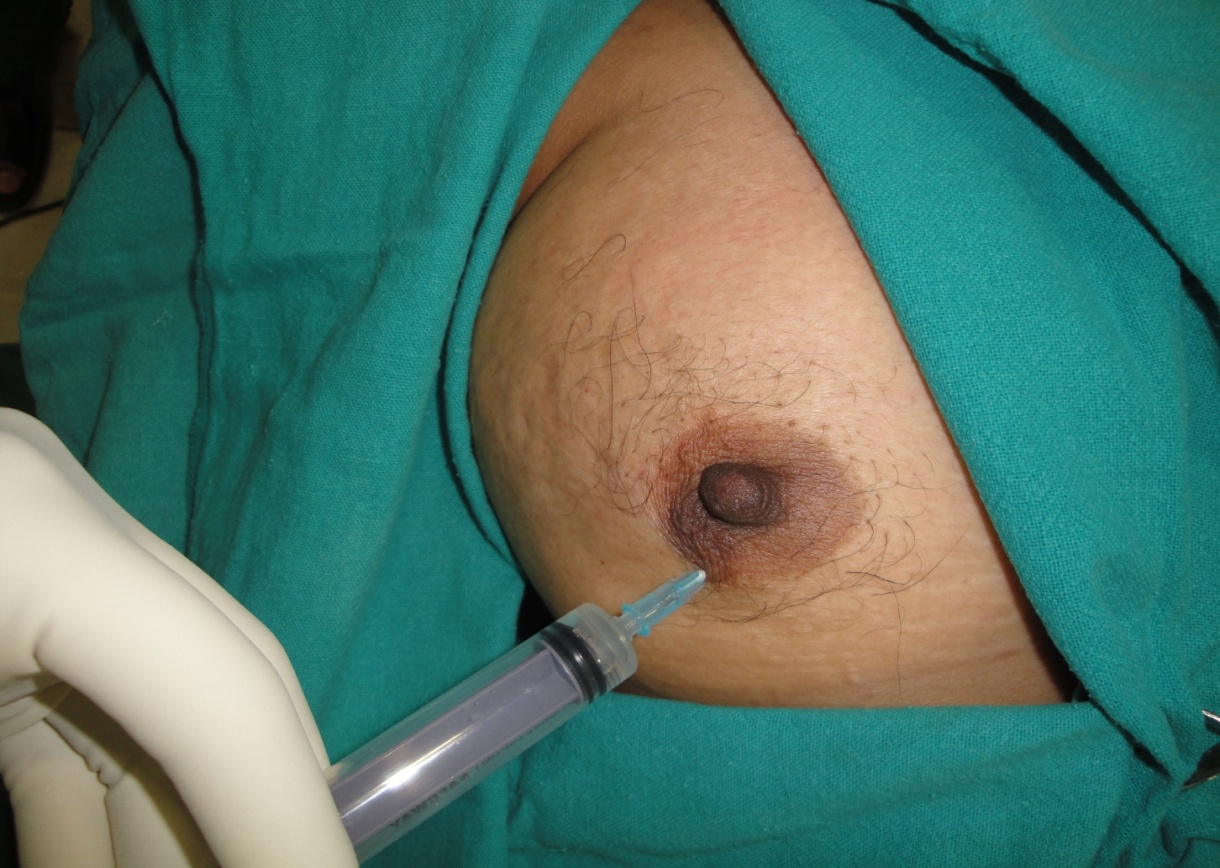


**Figure 4.3: Eutectic mixture of Figure 4.4: Injection of local**

**Ligocaine & Prilocaine applied anaesthetic agent**

**to NAC**


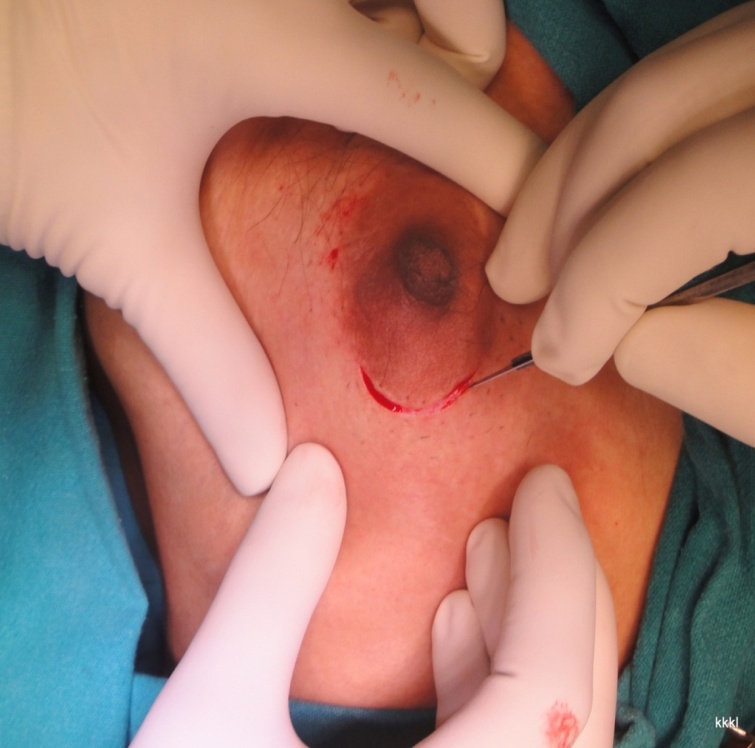
**
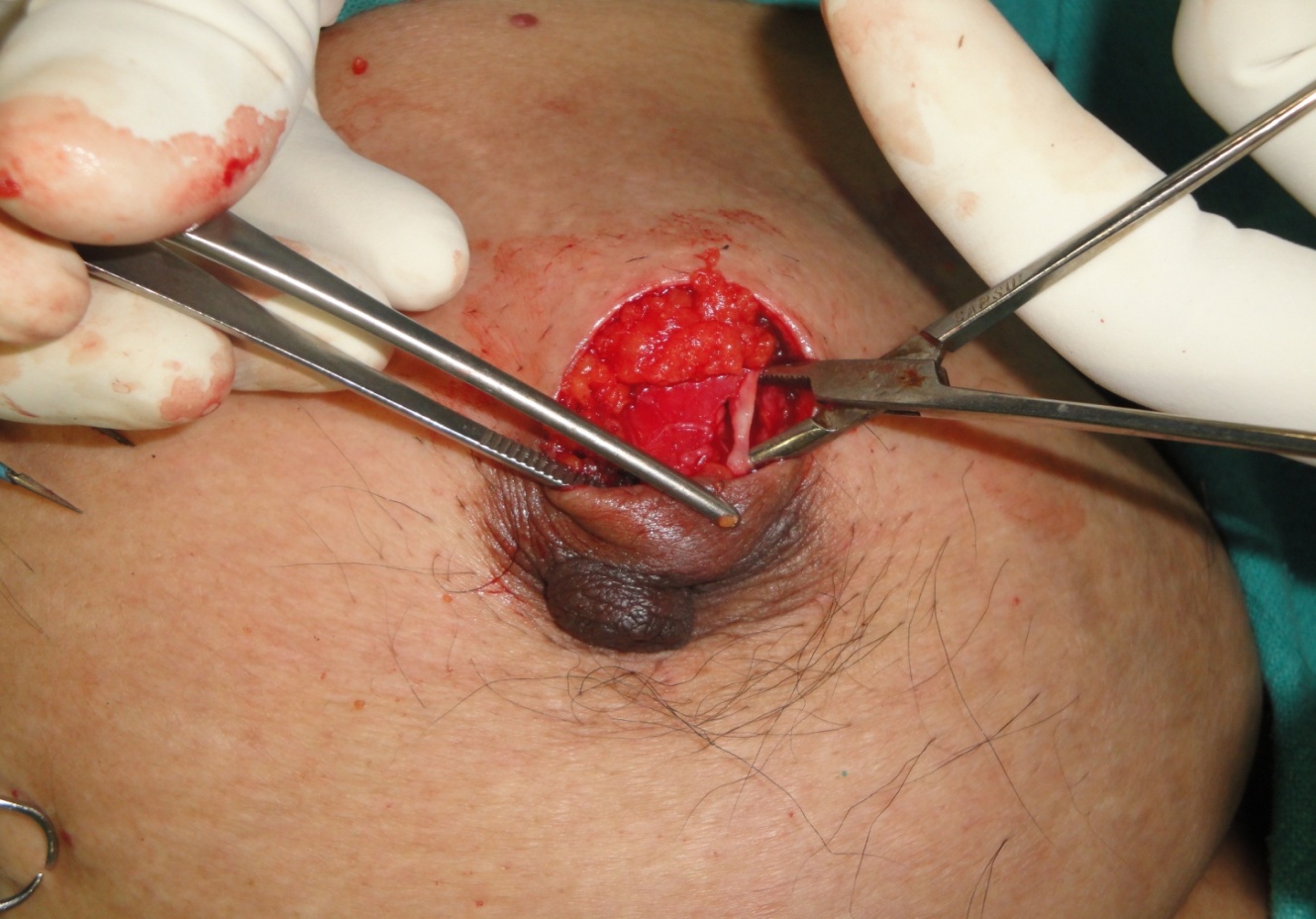
**

**Figure 4.5: Incision at the areola-skin Figure 4.6:Ductal cone with an**

**Junction over one-third of ectatic duct**

**areolar circumference**


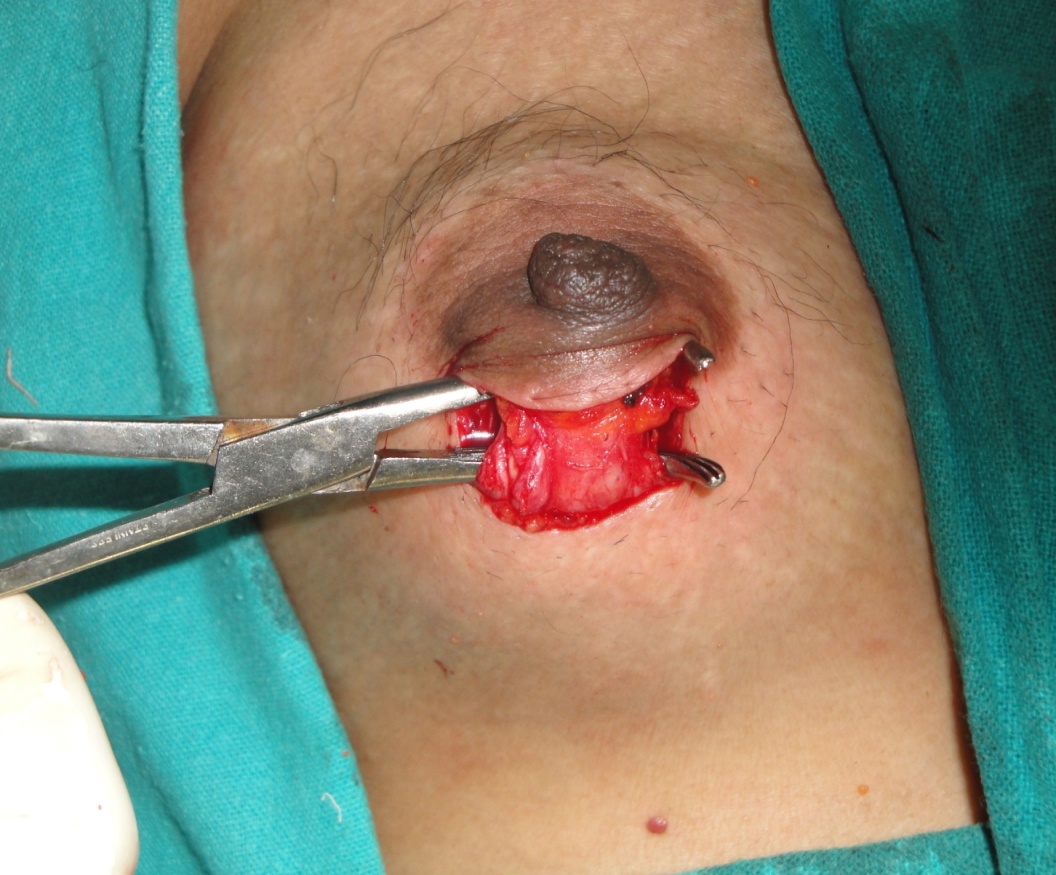

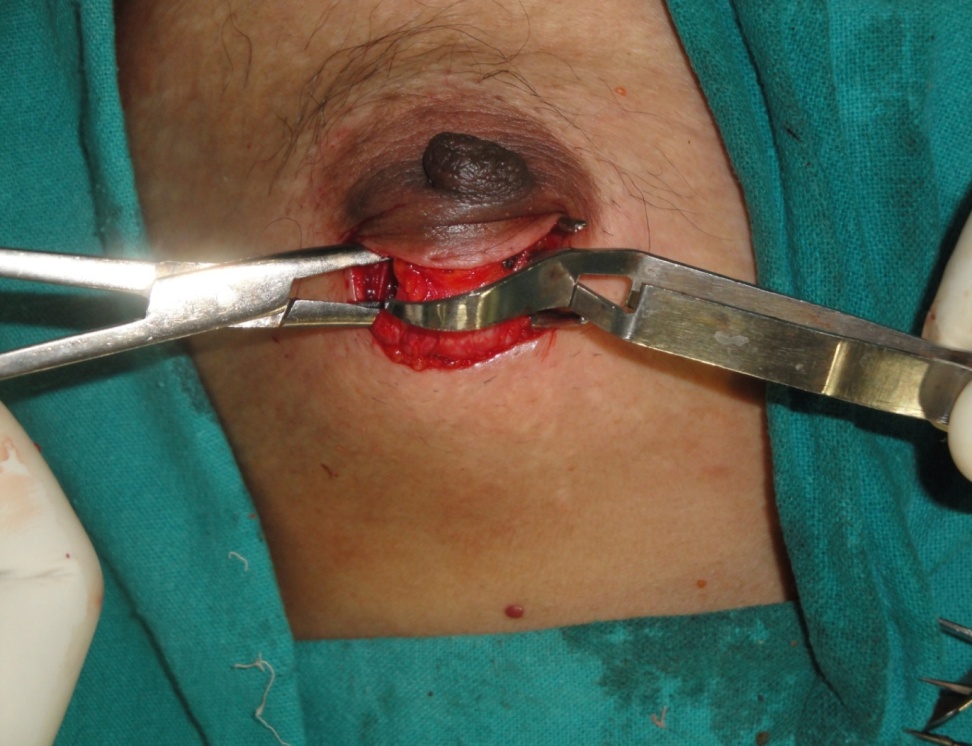


**Figure 4.7:Curved haemostat used Figure 4.8: Cross action towel clip**

**to create a subareolar tunnel behind clip applied across the ductal**

**the ductal cone cone**


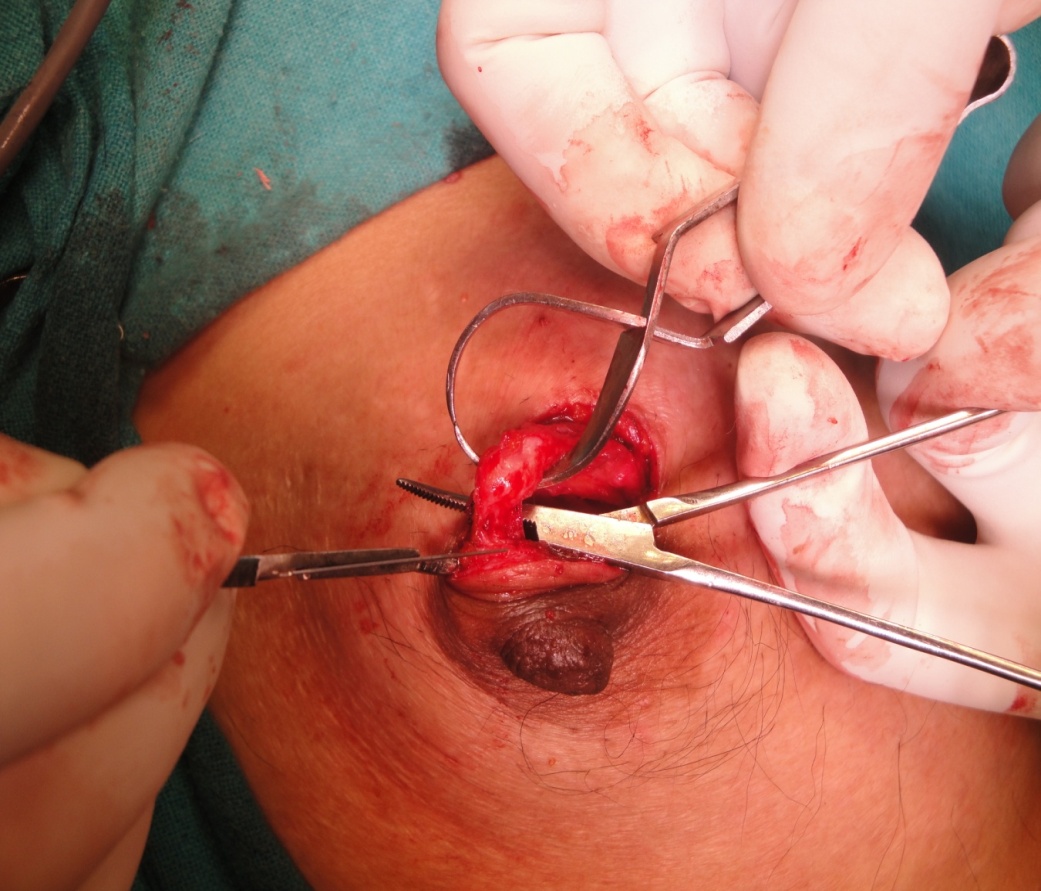

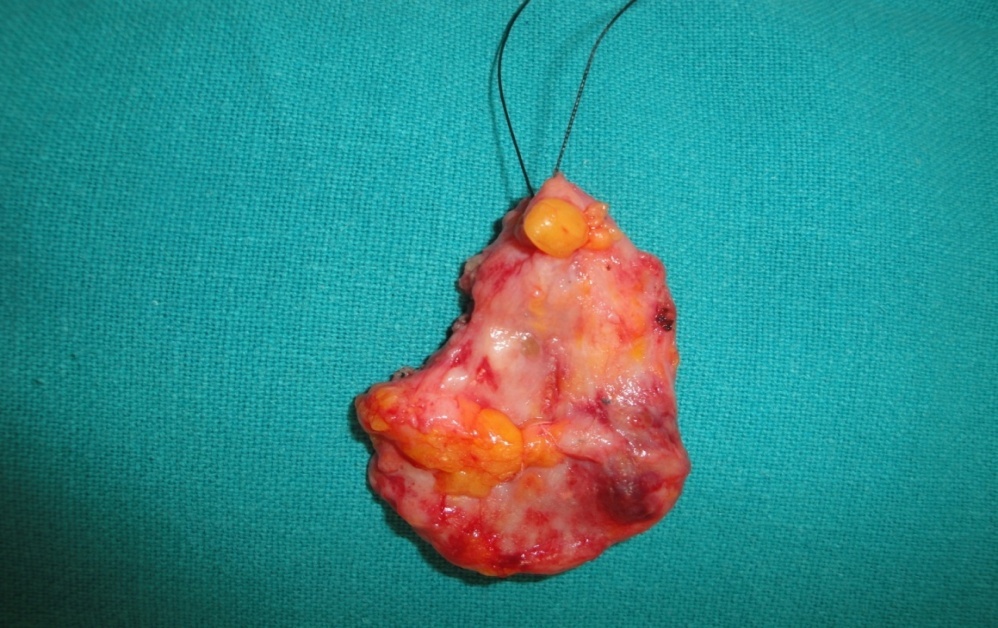


**Figure 4.9: Division of the ductal Figure 4.10: Marking of the**

**Cone specimen**

**
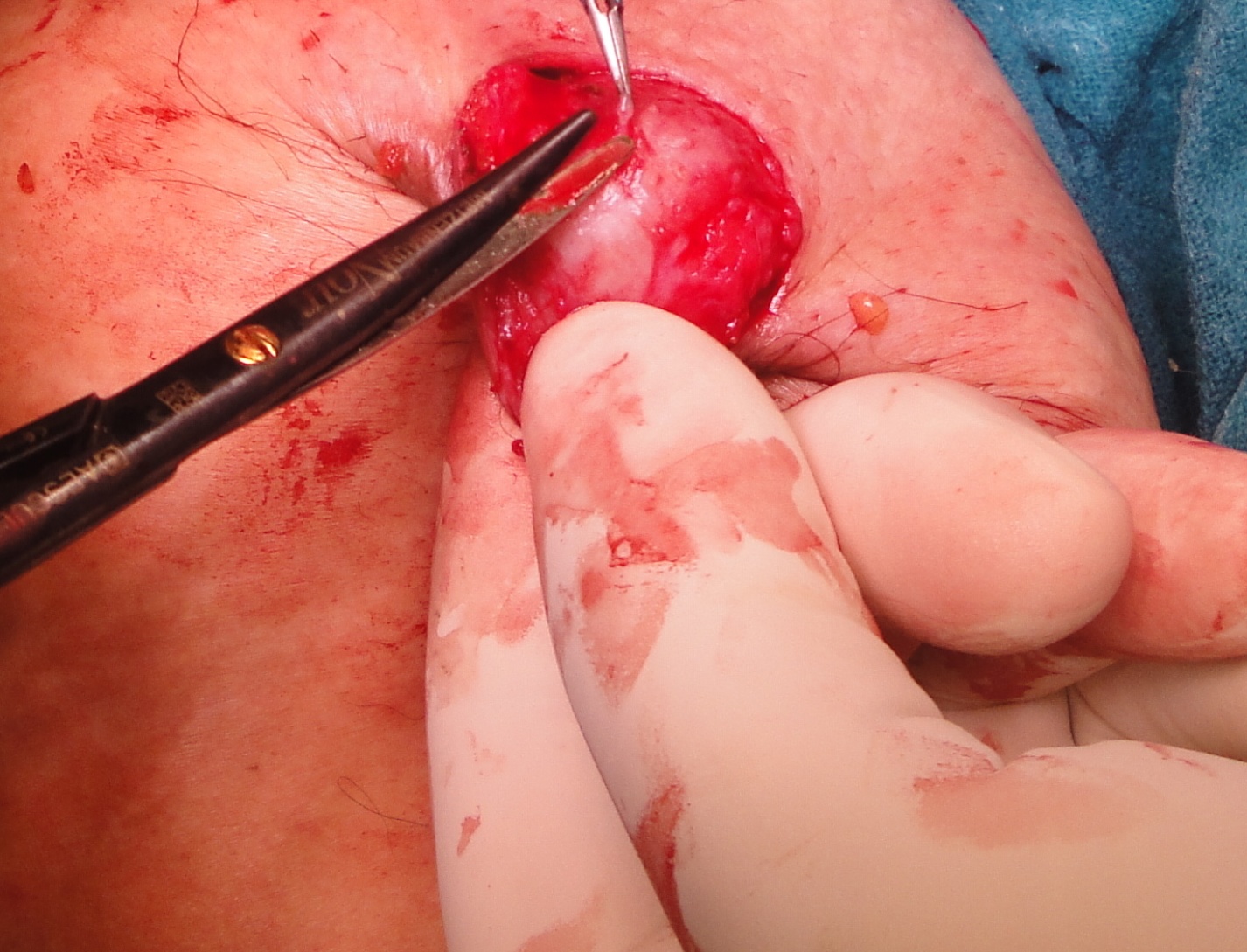

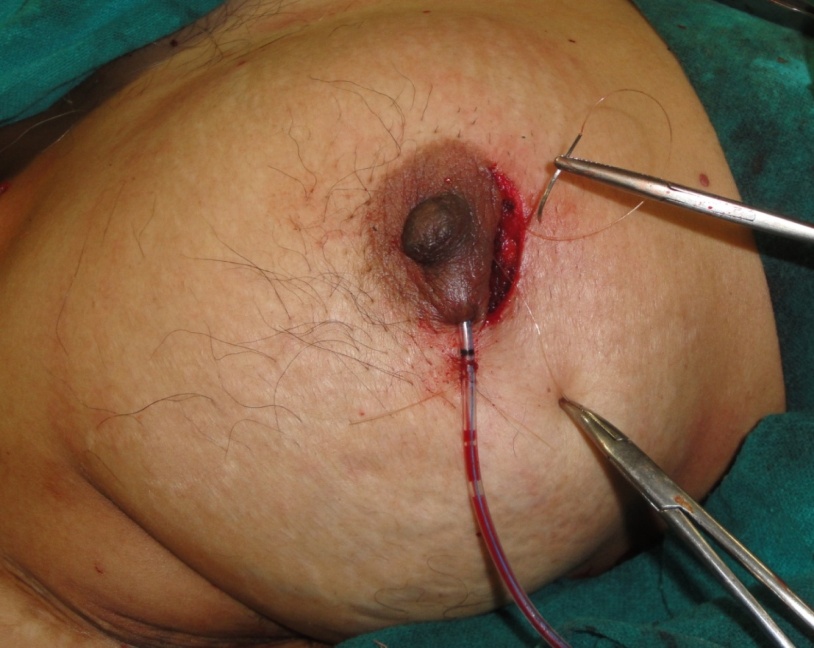
**

**Figure 4.11**: **Paring of terminal Figure 4.12: Closure with subcuticular**

**Portion of the major ducts Poliglecaprone 25 suture**

**and a suction drain**
