# Supplementary material for: Ultra structural changes occurring in duct ectasia and periductal mastitis and their significance in etiopathogenesis
Source: PLoS One. 2017 Mar 8;12(3):e0173216. doi: 10.1371/journal.pone.0173216 (PMC5342207; doi:10.1371/journal.pone.0173216)
Supplement: S2 File — (DOCX) [file pone.0173216.s002.docx]

**TRANSMISSION ELECTRON MICROSCOPY**

The normal mammary ducts were sampled from two patients who underwent reduction mammoplasty. These two ladies did not suffer from any nipple discharge and were premenopausal with regular periods. The mammary ducts from these two patients provided the information on the normal ducts. Ultrastructural study was performed in twenty patients. The tissues were processed by automatic tissue processor (figure 4.13, 4.14) after fixation with 2.5% glutaraldehyde in following methods,

1. Tissue was fixed in 2.5 % glutaraldehyde in 0.1 M sodium cacodylate buffer at 4^0^c, for overnight. Tissue was cut in to approximately 1 mm cubes for fixing.

2. Washed in 0.1 M buffer-1hour x 2. (or overnight at 4^0^c)

3. Post fixed in osmium tetroxide (OsO_4_) in 0.2 M buffer – 1 hour (equal quantities of 2 % aqueous OsO_4_ and 0.4 M buffer were mixed and used immediately.)

4. Rinsed in 0.2 M buffer 5 minutes x 2.

5. Dehydrated in 70 % ethanol- 20 minutes x 2.

6. Dehydrated in 90 % ethanol- 10 minutesx2.

7. Dehydrated in 100% ethanol- 20 minutes x 2.

8. Propylene oxide (1.2 epoxy propane)- 10 minutesx2.

9. Propylene oxide/ epoxy resin mixture (50/50)- 1 hour.

10. Epoxy resin- overnight.

11. Embedded in labeled capsules with freshly prepared resin.

12. Polymerize at 60^0^C – 48 hours.


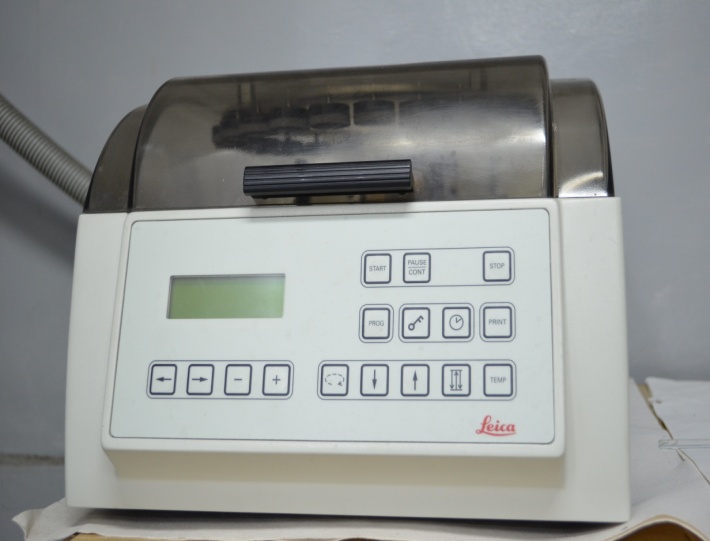


**Figure 4.13: Automated tissue processor**


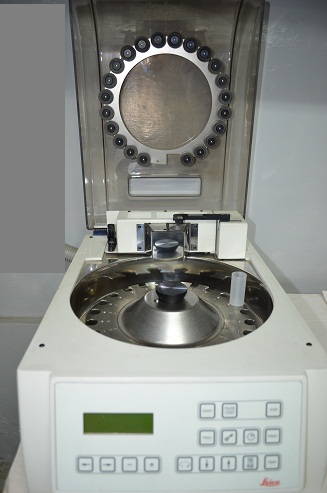


**Figure 4.14: Leica-automated tissue processor**

Semi-Thin Sectioning

(Allows selection of the appropriate tissue area before proceeding to E.M.)

1. Several resin sections were cut at approximately 1 micron using glass knives made by the knife maker (figure 4.14) and an ultra microtome (figure 4.15).

2. The sections were dried on to a glass slide on a hot plate at 80^o^C and then heated over flame for a few seconds to ensure adhesion.

3. The sections were stained with 1 % toulidine blue in 1 % borax solution for 1 minute at 80^0^C.

4. The stain was rinsed off with distilled water and the sections were dried and covered with a glass cover slip using a synthetic mounting medium such as D.P.X.

Ultra-Thin sectioning for Electron microscopy

1. The sections were cut in the same way as for thick sectioning but, using a diamond knife, with a Ultra microtome set to cut at around 70 nm using heat advance.

2. Sections were picked up on to the 300 mesh (300 squares) thin bar copper grid.

3. Then representative blocks were chosen for ultra-thin section, stained with uranyl acetate and lead citrate.

**Ultrastructural Examination**

Ultrastructural examination was performed on Philips Morgagni 268 transmission electron microscope (figure 4.16) and Technai G20 transmission electron microscope (4.17).


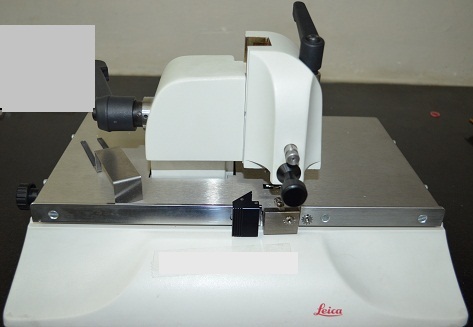


**Figure 4.15: Leica glass knife maker**


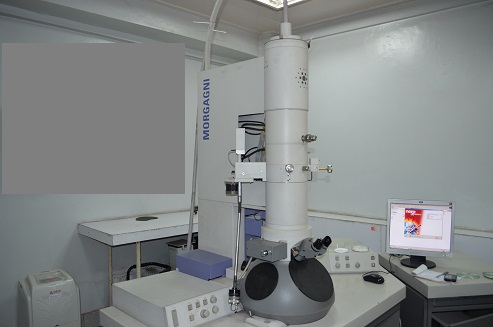


**Figure 4.16: Philips Morgagni 268 transmission electron microscope**


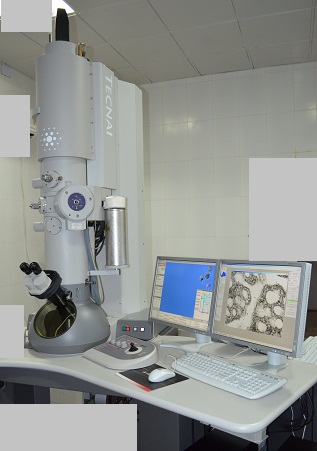


**Figure 4.17: Technai G20 transmission electron microscope**
