# Supplementary material for: Ultra structural changes occurring in duct ectasia and periductal mastitis and their significance in etiopathogenesis
Source: PLoS One. 2017 Mar 8;12(3):e0173216. doi: 10.1371/journal.pone.0173216 (PMC5342207; doi:10.1371/journal.pone.0173216)
Supplement: S3 File — (DOCX) [file pone.0173216.s003.docx]

The following are the photomicrographs of the various histopathological findings of the major mammary duct cone excisions done in the study.


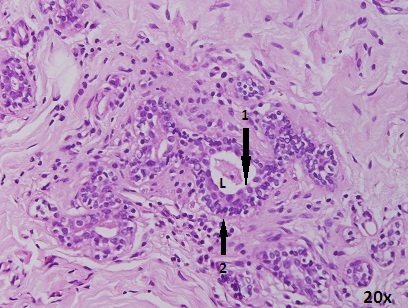


**Figure 5.4: Normal major duct-Histopathology (20x)**

This photomicrograph of normal ducts from a patient of reduction mammoplasty shows the normal sized duct with inner epithelial lining (arrow 1) and outer myoepithelial lining (arrow 2), L-lumen of the duct (H & E stain, x1000).

**
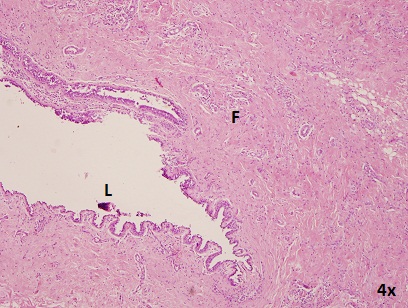
**

**Figure: 5.5 Duct Ectasia-Histopathology (4x)**

This photomicrograph of a case of duct ectasia shows the dilated duct in the low power field with marked periductal fibrosis with mild chronic inflammation. (H & E stain, x200).


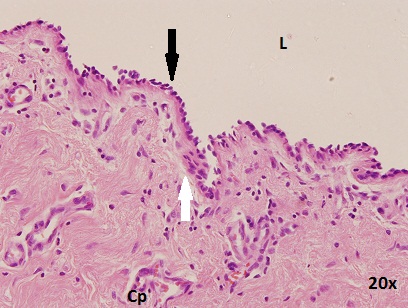


**Figure: 5.6 Duct Ectasia-Histopathology (20x)**

Higher magnification of the dilated duct in this photomicrograph shows focal denudation of the epithelial cells (black arrow), focal proliferation of the myoepithelial cells (white arrow), mild chronic inflammation (lymphocytes and a few plasma cells) and a few dilated capillaries (Cp) in the periductal tissue. (H & E stain, x1000).


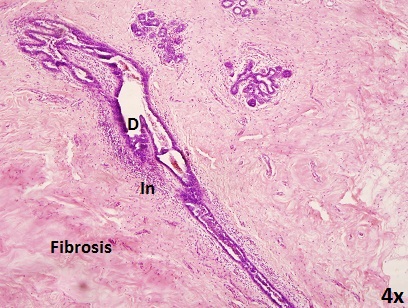


**Figure: 5.7 Histopathology Of Periductal Mastitis (4x)**

This photomicrograph shows focal dilatation of the duct with periductal fibrosis and dense periductal inflammation (H & E stain, x200).


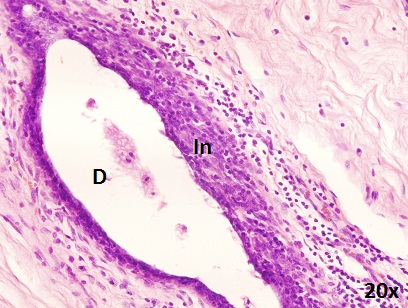


**Figure: 5.8 Histopathology Of Periductal Mastitis (20x)**

High power of the focally dilated segment of the duct seen in the photomicrograph shows dense chronic inflammation (In) with intact epithelial cells lining the duct seen with plasma cell predominance (H&E stain,x1000).

**
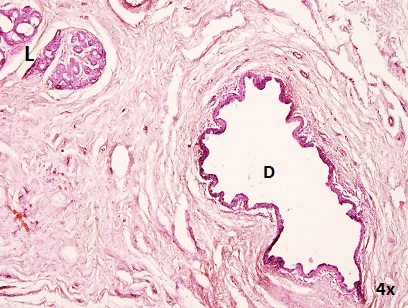
**

**Figure: 5.9 Histopathology of duct ectasia with periductal mastitis (4x)**

This photomicrograph shows dilated duct (D) with periductal inflammation and the adjacent lobule (L) shows features suggestive fibrocystic disease (focal apocrine metaplasia) (H&E, x200)


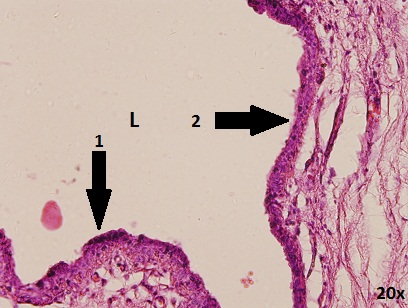


**Figure: 5.10 Histopathology of duct ectasia with periductal mastitis (20x)**

This photomicrograph shows the dilated duct with preserved epithelial lining (1) in most of the places with focal areas of epithelial denudation (2). L- lumen of the duct (H&E,x1000)


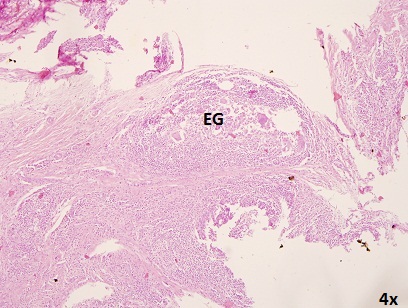


**Figure: 5.11 Histopathology of tuberculous mastitis (4x)**

This photomicrograph shows epitheloid granuloma (EG) in the periductal tissue, surrounded by dense lymphocytic infiltration in a patient with tuberculous mastitis (H&E, x200).


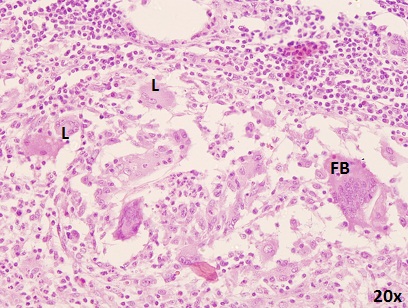


**Figure: 5.12 Histopathology of tubeculous mastitis (20x)**

This photomicrograph shows high power of the epitheloid granuloma with langhan giant cells (L) and foreign body giant cells (FB) in the periductal tissue with dense lymphocytic infiltration (H&E, x1000).

**Plasma cell percentage in PDM, PDM with DE and FCD with PDM with or without DE**

In PDM, the percentage of plasma cells per high power field were from 32 to 44% with a mean of 38%. In DE with PDM, the plasma cell percentage was from 16 to 28 % with a mean of 22%. In FCD with PDM with or without DE, the plasma cell percentage ranged from 14 to 26% with a mean of 20%.

This shows that plasma cell response is relatively higher in pure PDM than in association with DE or fibrocystic disease.

**Normal Major Mammary Duct – Transmission Electron Microscopy**

The major mammary duct in the electron microscope shows a continuous epithelial layer, single to double layered with myoepithelial cells lining the duct in a discontinuous manner (Figure 5.13 and 5.14). Two types of epithelial cells lining the major mammary duct could be appreciated namely, the dark epithelial cells and the light epithelial cells (Figure 5.15). The dark epithelial cells are rich in electron dense ribosomes (Figure 5.16). In the interepithelial junctions there a terminal bars (T bars) which are seen electron dense (Figure 5.17). The myoepithelial cells show cytoplasmic extensions into the periductal tissue and may contain occasional lipid vesicles(Figure 5.18).

**Duct ectasia – Transmission Electron Microscopy**

There is flattening of the duct epithelium in the dilated duct at places and focal loss of microvilli of the duct epithelium are present (Figure 5.19). Focal areas of epithelial denudation with intraluminal epithelial debris are seen (Figure 5.20). Multiple membrane bound dark and light vesicles are seen in the cytoplasm of the duct epithelial cells (Figure 5.21, 5.22 and 5.23). The duct epithelial cells at places show elongated nucleus with plenty of intracytoplasmic fibrillary structures suggestive of epithelial mesenchymal transformation (Figure 5.24 and Figure 5.25). There is focal distortion of terminal bar and widening of the inter-epithelial junction present (Figure 5.26). At places the duct epithelial cells show numerous cytoplasmic projections (Figure 5.27 and Figure 5.28)

Small ducts (ductules) (Figure 5.29) also show similar epithelial changes as that of the major ducts. Duplication of the basal lamina of the duct (Figure 5.30 and 5.31) is seen at places. Marked periductal collagenisation is seen (Figure 5.32 and 5.33). At places, in the periductal tissue, dilated lymphatic vessels are seen (Figure 5.34 and 5.35).

**Periductal mastitis – Transmission Electron Microscopy**

Periductal infiltration by the macrophages (Figure 5.36), lymphocytes and plasma cells (Figure 5.37 and 5.38) from the blood vessels are seen. The epithelial lining is intact with preserved microvilli (Figure 5.39). The epithelial cells are hypertrophic in some ducts indicative of active epithelial proliferation (Figure 5.40). The inter epithelial junctions are intact with preserved T bars. The epithelial cell cytoplasm shows dilated mitochondria with loss of cristae and prominent endoplasmic reticulum suggestive of injury pattern (Figure 5.41). There are numerous pinocytic vesicles in the cytoplasm of the epithelial cells suggestive of active secretion by the duct epithelial cells (Figure 5.42). The myoepithelial cells show dense inclusion bodies (Figure 5.43). There is neovascularisation secondary to inflammation as there are new vessels with plumpy endothelial cells being seen in the periductal tissue (Figure 5.44). The fibroblast cells show plenty of vesicles with abundant collagen granules suggestive of active collagenisation (Figure 5.45). The following are the electro micrographic pictures showing the above mentioned findings.

**Normal Major Mammary Duct – Transmission Electron Microscopy**

**
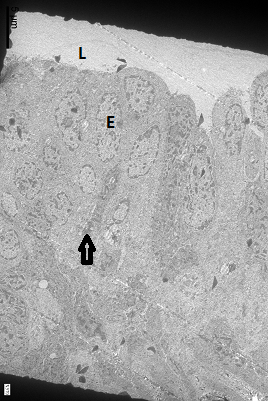
**

**Figure 5.13**

This electro micrograph shows transverse section of the major duct with continuous epithelial layer (E) with underlying myoepithelial cells (denoted by arrow) which are discontinuous. L – Lumen of the duct. (x570)


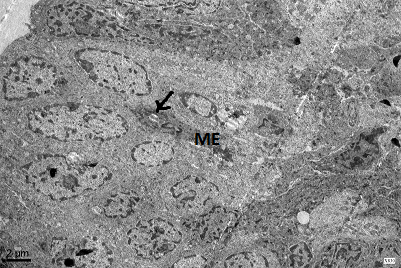


**Figure 5.14**

Higher magnification of the transverse section of the duct shown in the electro micro graph (fig.5.13). The myoepithelial cell (ME) shows occasional lipid bodies in the centre denoted by the arrow. (x830)


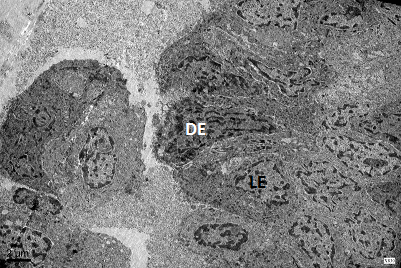


**Figure: 5.15**

The dark and light epithelial cells of the duct are seen with different electron densities are seen in this electromicrograph of the normal duct cross section. (x830)


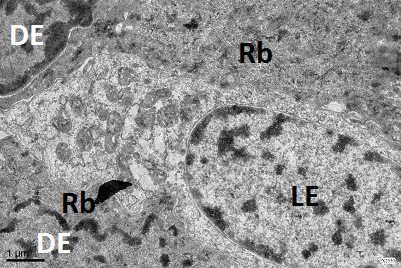


**Figure: 5.16**

The above electro micrograph is a higher magnification of dark (DE) and light epithelial cells (LE), with dark epithelial cells showing dense distribution of ribosomes (Rb).

**
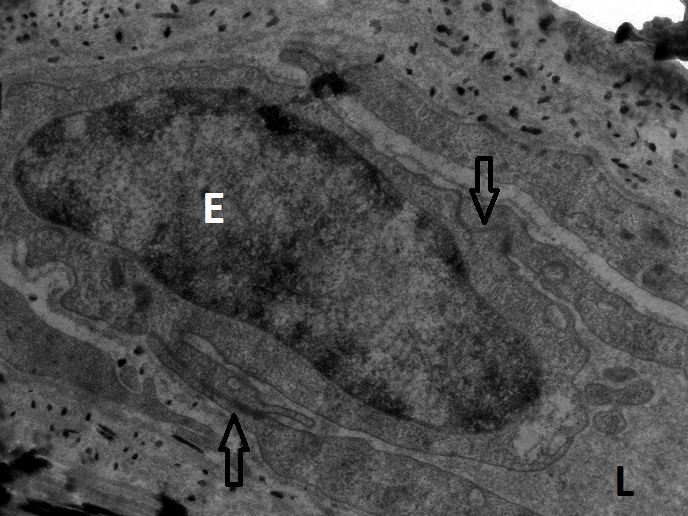
**

**Figure: 5.17**

This electro micrograph of a duct in a patient with duct ectasia shows the presence of T bars in the inter epithelial junctions (as depicted by the two arrows). E- epithelial cell, L- lumen of the duct. (x5000)

**
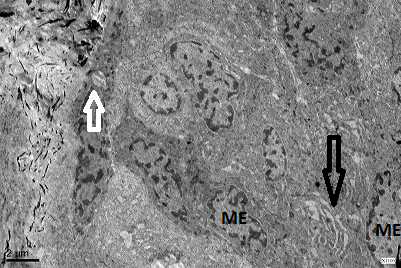
**

**Figure: 5.18**

The myoepithelial cells (ME) seen in this electro micrograph shows multiple extensions (black arrow) into the periductal tissue forming complex arborising patterns. Few lipid vacuoles (white arrow) in the cytoplasm of the myoepithelial cells are seen. (x1100)

**Duct Ectasia-Transmission Electron Microscopy**


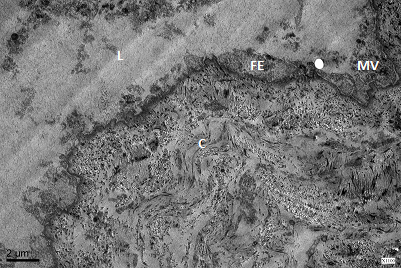


**Figure: 5.19**

The above electro micrograph shows portion of the dilated duct with areas of flattened epithelium (FE) with focal presence of microvilli(MV) and marked periductal collagenisation(C). (x1100)


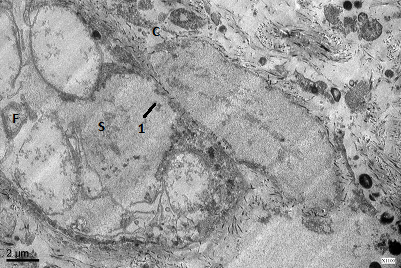


**Figure 5.20**

Low power electro micro graphic view of a dilated duct with periductal tissue showing focal denudation (1) of lining epithelium with intraluminal secretions(S) containing epithelial fragments (F). Around the duct there is collagen deposition. (x1100)


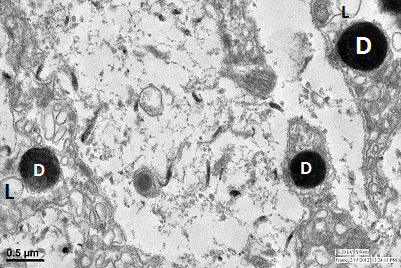


**Figure 5.21**

Magnified portion of the epithelial cell in this electro micrograph showing membrane bound dark (D) and light (L) vesicles in the cytoplasm. (x5000)


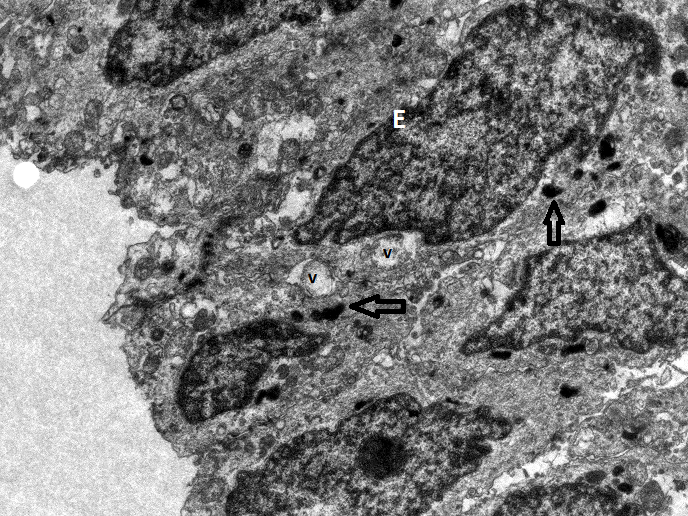


**Figure:5.22**

The above electro micrograph is the magnified picture of the epithelial cells shown in figure 5.36. The epithelial cells show large number of vacuoles (V) and dense bodies (indicated by the arrows). E – epithelial cell. (x2000)


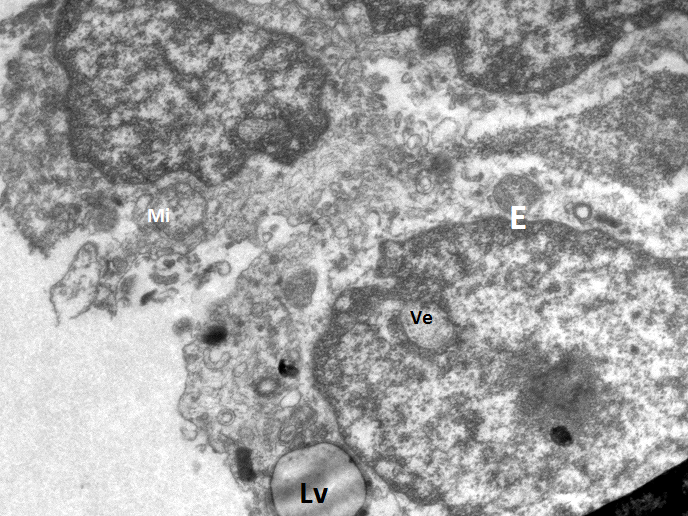


**Figure: 5.23**

The portion of the epithelial cell (E) shown in the above electro micrograph shows intracytoplasmic lipid vacuole (Lv) and an intranuclear membrane bound vesicular inclusion. The cytoplasm of the epithelium also shows dilated mitochondria (Mi). (x4000)


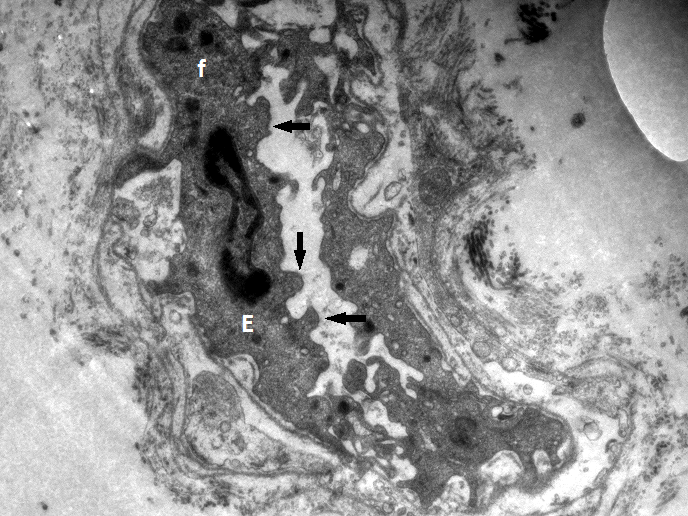


**Figure: 5.24**

The duct epithelial cell (E) shown in the electro micrograph has elongated nucleus, intracytoplasmic fibrils (f) and intraluminal projections (indicated by the arrows) of the cytoplasm suggestive of mesenchymal transformation of the epithelial cell. (x3200)


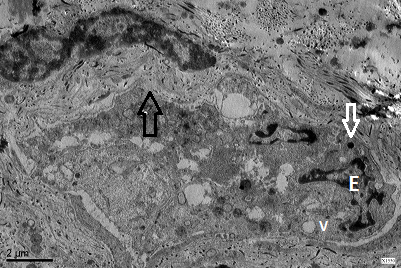


**Figure 5.25**

This electromicrograph shows cross section of a small dilated duct. Epithelial denudation with disruption of the basal lamina has been shown in black arrow. The duct epithelial cell (E) shows elongate nucleus with plenty of intracytoplasmic fibrillary structure, vacuoles (V) and dense body (shown by a white arrow) suggestive of epithelial mesenchymal transformation. (x1550)


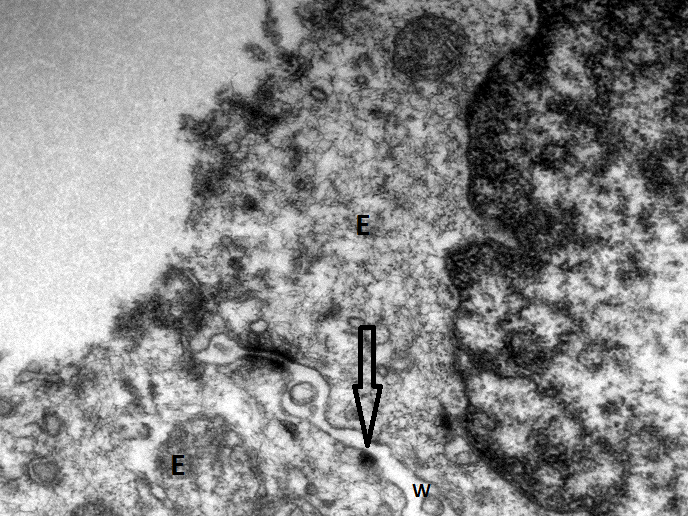


**Figure: 5.26**

This electro micrograph shows further higher magnification of the epithelial cells with the inter epithelial junction shown in figure 5.39. It depicts focal distortion of terminal bar (indicated by the arrow) and widening of the interepithelial junction (W). E –epithelial cells. (x8000)


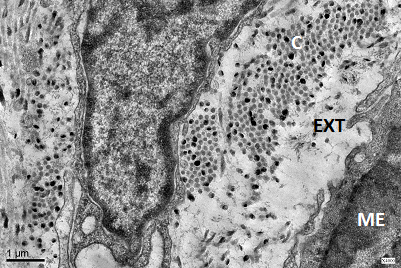


**Figure 5.27**

The myoepthelial cell (ME) surrounded by dense collagen fibres (C) with epithelial extensions (EXT) projecting into the periductal tissue is seen in the above electro micrograph. (x4000)


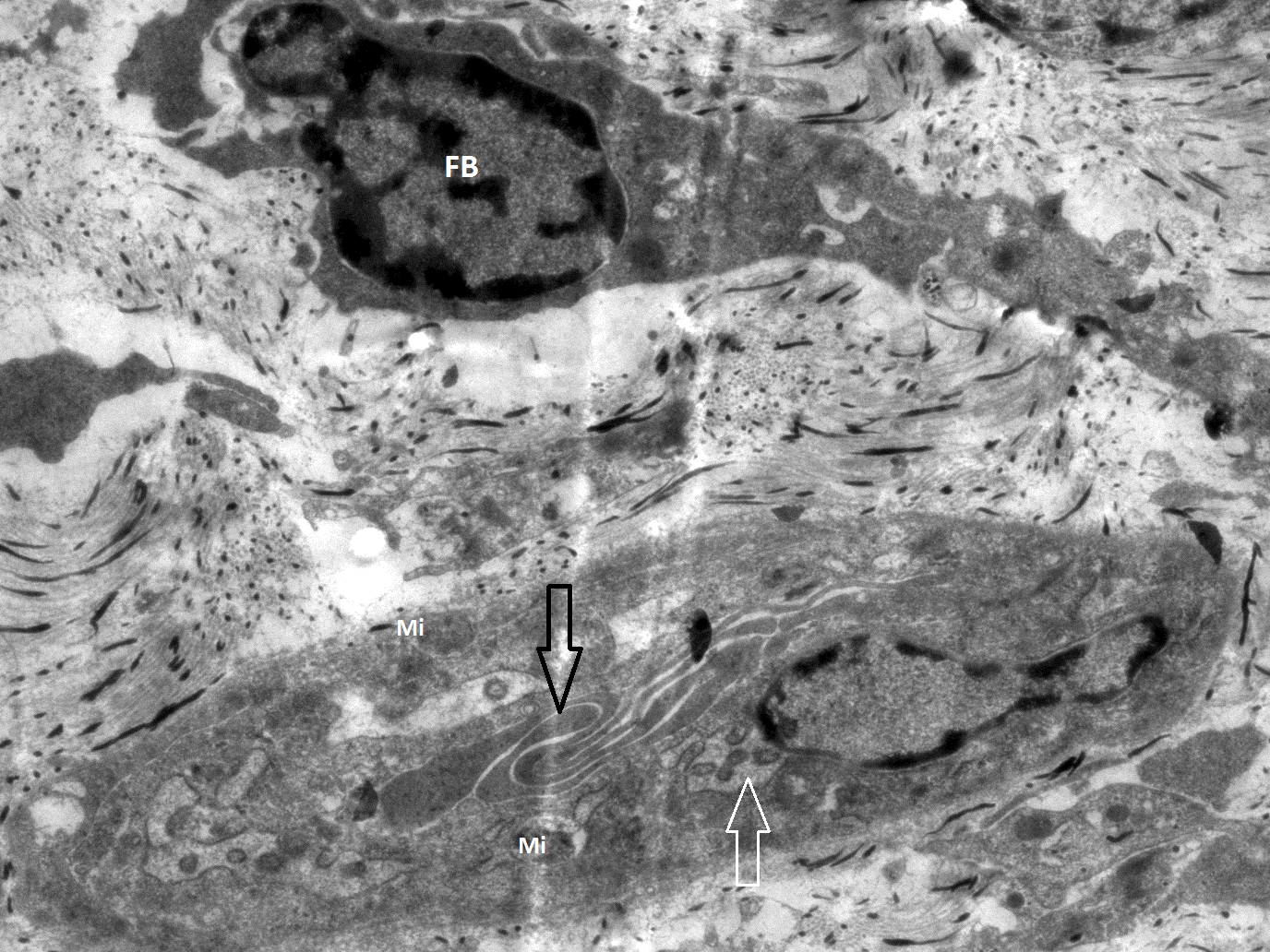


**Figure 5.28**

This electro micrograph shows transverse section of a dilated duct with epithelial cell containing microvilli in the luminal side (indicated by the white arrow). The epithelial also has a long cytoplasmic processes projecting into the lumen and large dilated mitochondria with cristae suggestive of injury/reactive pattern. (x2000)


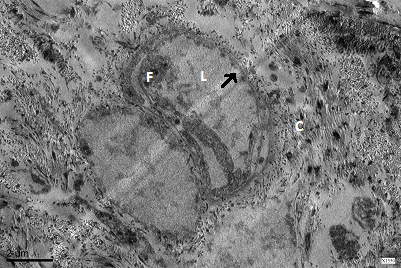


**Figure 5.29**

Ductule in the above mentioned electromicrograph shows focal area of epithelial denudation (arrow) and with intraluminal(L) denuded epithelial fragments(F) and extensive periductal collagen deposition(C). (x1550)


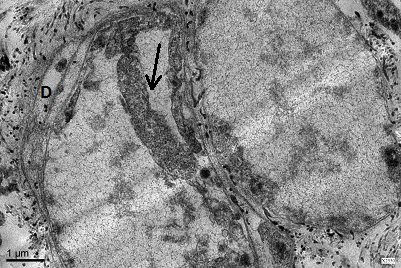


**Figure 5.30**

This electro micrograph is the higher magnification of the ductule shown in the figure 5.29 showing lifting of the epithelium from the basal lamina (arrow) and duplication of the basal lamina(D). (x2550)


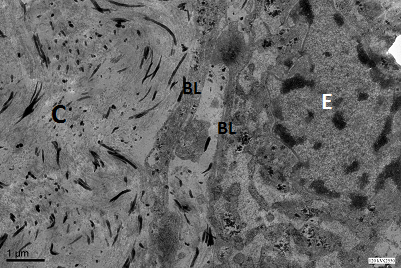


**Figure: 5.31**

The basal lamina (BL) adjacent to the duct epithelial cell (E) in the above electro micrograph shows duplication. C – collagen. (x2550)


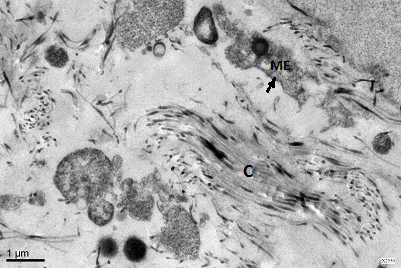


**Figure 5.32**

This electro micrograph shows periductal region with collagen fibrils interspersed between the myoepithelial cells. (x2550)





**Figure 5.33**

The above electro micrograph shows periductal tissue filled with thick and thin collagen fibres indicative of active collagenisation. (x2550)


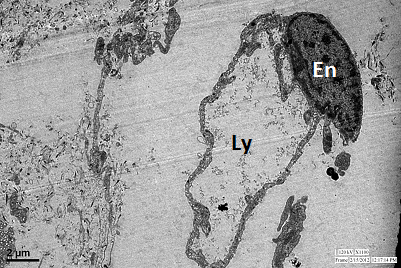


**Figure 5.34**

Dilated lymphatic vessel (Ly) with the endothelial cell (En) in the periductal tissue seen in this electro micrograph. (x1100)


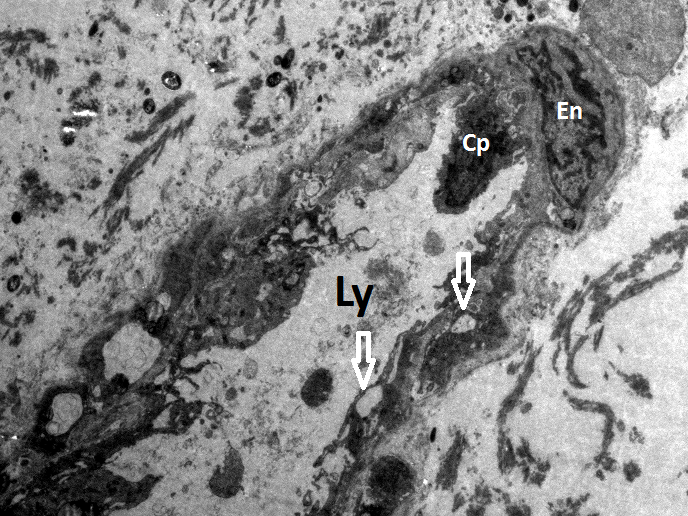


**Figure: 5.35**

This electro micrograph shows a dilated lymphatic vessel (Ly) in the vicinity of the periductal tissue. The endothelial cell (En) shows large intracytoplasmic vacuoles (indicated by the arrows) and cytoplasmic projection (Cp) into the lumen. (x1000)

**Periductal mastitis – Transmission Electron Microscopy**

**
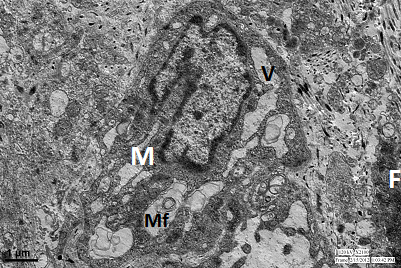
**

**Figure 5.36**

This electro micrograph show a Macrophage (M) in the periductal tissue with cytoplasmic vacuoles (V) and myelin figure like structures (Mf). F-fibroblast. (x2100)


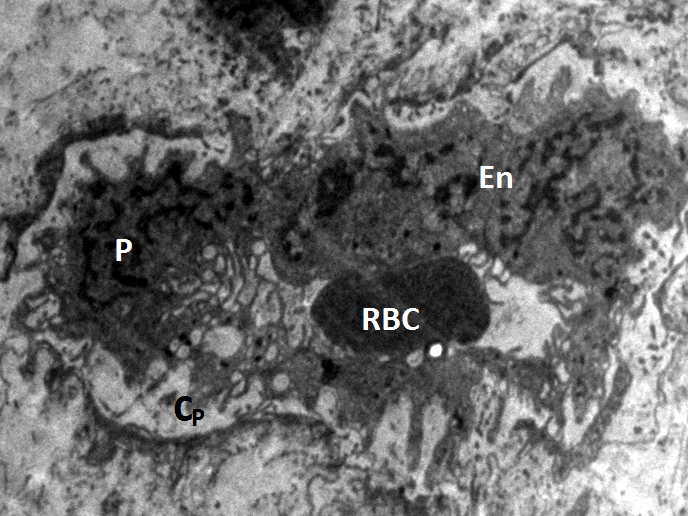


**Figure: 5.37**

The electro micrograph in Figure 5.37 shows capillary (Cp) in the periductal tissue showing the presence of plasma cell (P) and RBC suggestive of plasma cell infiltration from the blood stream. (x1600)


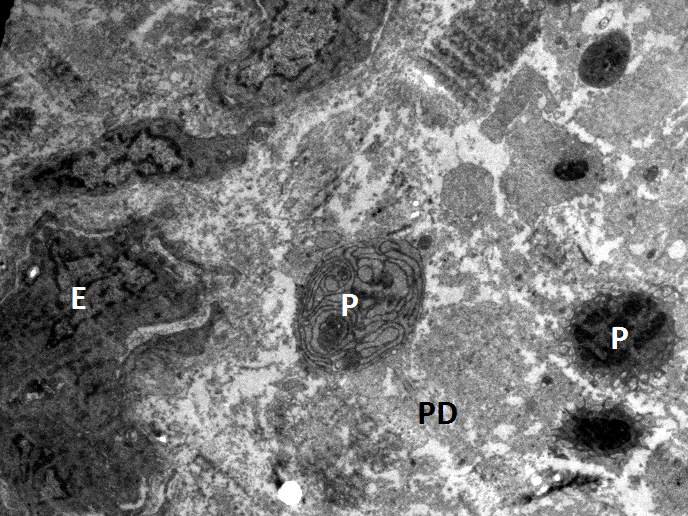


**Figure: 5.38**

Figure 5.38 shows the infiltration of the periductal tissue by the plasma cells. (x1000) En – endothelial cell, DP- degenerated plasma cell, E- epithelial cell, PD- periductal tissue.


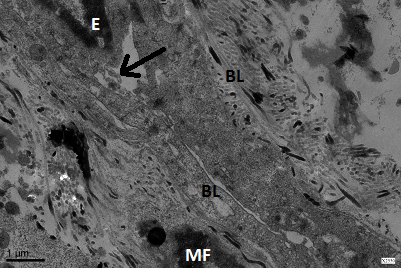


**Figure 5.39**

This electro micrograph shows the longitudinal section of a non dilated duct with intact epithelial lining. The epithelium (E) shows microvilli on the surface (arrow). The myofibroblast cell (MF) is seen adjacent to the basal lamina (BL). (x2550)


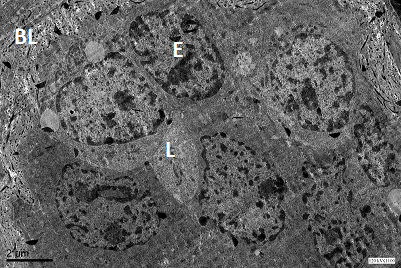


**Figure 5.40**

The above mentioned electro micrograph shows transverse section of the duct lined by hypertrophic epithelial cells (E) almost filling the entire lumen (L) indicative of epithelial proliferation. BL – Basal Lamina (x1100)


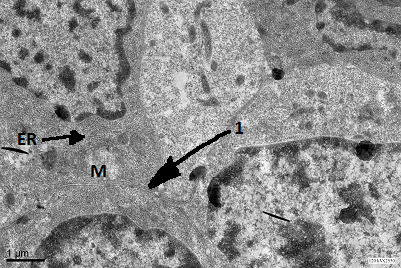


**Figure 5.41**

The arrow in the above mentioned electro micrograph represents the inter epithelial junction. The epithelial cell shows dilated mitochondria with loss of cristae (M) and prominent endoplasmic reticulum (ER). (x2550)


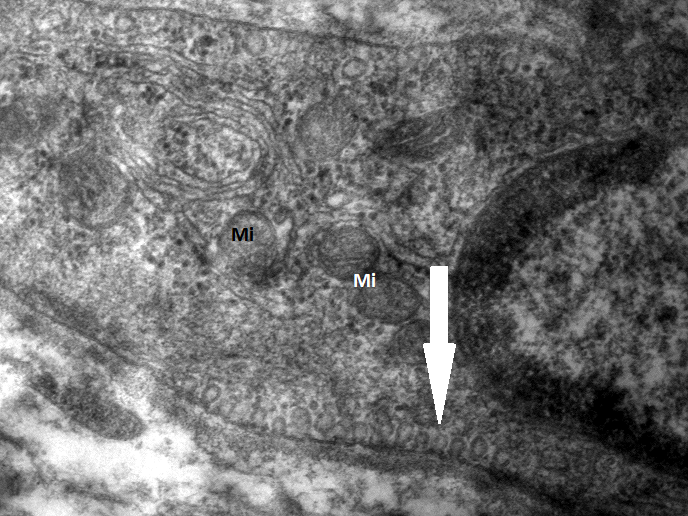


**Figure: 5.42**

The ultrastructure of the portion of the duct epithelial cell depicted in this electro micrograph shows numerous pinocytic vesicles (some fusing with the membrane – depicted by the arrow) suggestive of active secretion by the epithelial cells. The cytoplasm also shows large mitochondria with dilated cristae. (x13000)


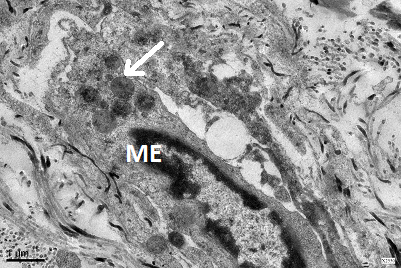


**Figure 5.43**

The above shown electro micrograph of the myoepithelial cell shows dense inclusion bodies (DB) in the cytoplasm. (x2550)


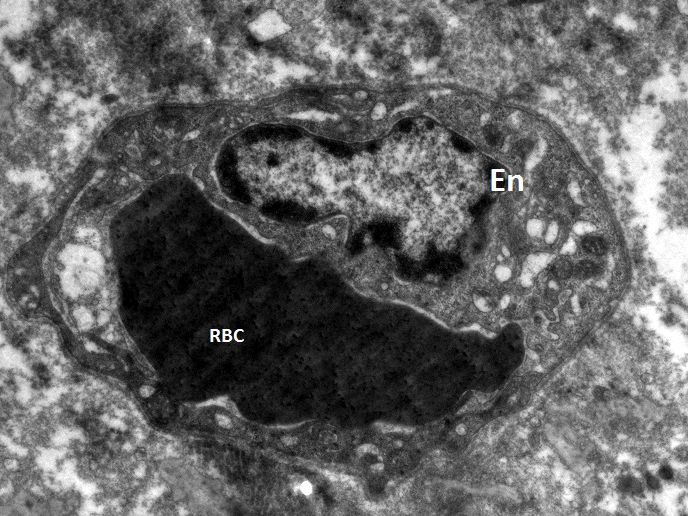


**Figure: 5.44**

This electro micrograph shows capillary in the periductal tissue with plump endothelial cell and an inspissated red blood cell. This is suggestive of neovascularisation secondary to inflammation. (x3200)


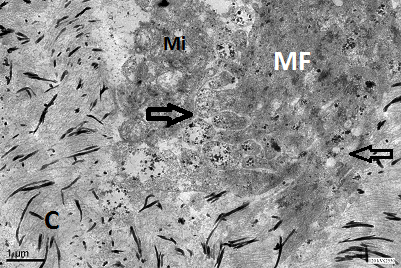


**Figure: 5.45**

This electro micrograph shows a portion of a myofibroblast cell(MF) with lot vesicles (indicated by the arrows) containing collagen granules suggestive of active collagenisation. The cell also shows multiple dilated mitochondriae (Mi). All these changes are indicative of exaggerated cell function.C – Collagen fibres. (x1550)


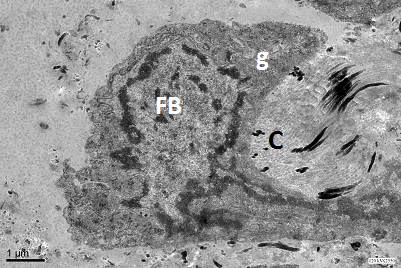


**Figure: 5.46**

The myofibroblast (MF) in the above mentioned electro micrograph shows dense glycogen granules (g) with active collagenisation (C). (x2550)

**IMMUNOHISTOCHEMISTRY (IHC)**

**IHC FOR VIMENTIN (A MESENCHYMAL MARKER)**

The epithelial cells in case of DE have shown focal areas of uptake of vimentin (Figure-5.48), which is not seen in cases of periductal mastitis (Figure-5.49).


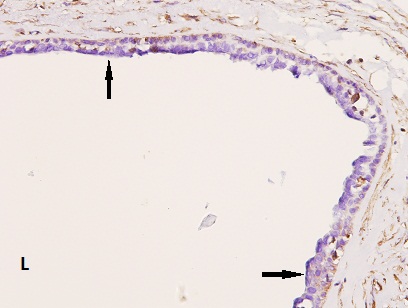


**Figure: 5.48**

(The arrows indicate the focal uptake of vimentin by the epithelial cells.)

**
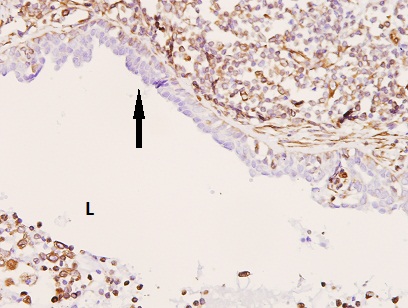
**

**Figure: 5.49**

L-lumen of the duct.
